# Definition of Skills and Roles of the Digestive Endoscopy Nurse: Italian Consensus of ANOTE-ANIGEA

**DOI:** 10.1097/SGA.0000000000000887

**Published:** 2025-09-23

**Authors:** Daniele Napolitano, Maria Luigia Candela, Mirko Gaggiotti, Laura Turchini, Gabri Bertaglia, Angela Nicoletta Minenna, Monia Valdinoci, Teresa Iannone, Nazario Munno, Giorgio Iori, Antonella Giaquinto, Alessandra Guarini, Giulio Petrocelli, Angelo Zullo

**Affiliations:** **About the authors:** Daniele Napolitano, MSN, RN, CEMAD-Fondazione Policlinico Gemelli IRCCS, Roma, Italy.; Maria Luigia Candela, PhD, RN, Endoscopia, Policlinico Campus Biomedico, Roma, Italy.; Mirko Gaggiotti, RN, Endoscopic Section of Gastroenterology and Hepatology, Perugia General Hospital and University of Perugia, Perugia, Italy.; Laura Turchini, RN, CEMAD-Fondazione Policlinico Gemelli IRCCS, Roma, Italy.; Gabri Bertaglia, MSN, RN, Presidio Ospedaliero Schiavonia AULSS 6 Euganea, Padova, Italy.; Angela Nicoletta Minenna, RN, ASL BA – P.O. San Paolo, Bari, Italy.; Monia Valdinoci, RN, Azienda Ospedaliero Universitaria Careggi – Firenze, Italy.; Teresa Iannone, RN, U.O. di Gastroenterologia ed Endoscopia Digestiva – ASP 5 Ospedale di Polistena, Reggio Calabria, Italy.; Nazario Munno, RN, ASST Brianza Ospedale “Pio XI” di Desio, Milano, Italy.; Giorgio Iori, RN, Gastroenterology and Digestive Endoscopy Unit, Azienda USL-IRCCS Reggio nell’Emilia, Reggio Emilia, Italy.; Antonella Giaquinto, RN, Endoscopy Unit, Tor Vergata University, Rome, Italy.; Alessandra Guarini, RN, Gastroenterology and Digestive Endoscopy, “Nuovo Regina Margherita” Hospital, 00153 Rome, Italy.; Giulio Petrocelli, RN, Digestive and Interventional Endoscopy Unit, ASST Niguarda, Milan, Italy.; Angelo Zullo, PhD, MD, Gastroenterology and Digestive Endoscopy, “Nuovo Regina Margherita” Hospital, 00153 Rome, Italy.; ANOTE-ANIGEA STUDY GROUP: Andrea Marino, Pierangelo Simonelli, Anna Sabina D’Inca, Tamara dal Zot, Giovanni Fiorito, Sandro Simonelli, Bernardo Baiocco, Roberta Cesaro, Rossella Varotto, Luigina Schirru, Giorgia Chiara Zaniboni, Elisa Schiavoni, Elsa Vitale, Alice Aliberti, Lorenzo Maria Vetrone, Marta Pastorelli, Fabio Grilli, Arianna Parrella, Arianna Povoli, Fabio Antonio Sacco, Giuseppe Romeo, Letteria Consolo, Tommaso Tassinari, and Chiara Massari.

## Abstract

Endoscopy nurses work in all phases of endoscopic procedures, guaranteeing privacy, respect for dignity, comfort, and safety by applying specific theoretical knowledge and practical skills. However, in several countries, a structured training process in this specific area is lacking. This document defines the skills and roles of nurses working in the endoscopy and gastroenterology units in Italy. The National Association of Endoscopic Technique Operators - National Association of Gastroenterology Nurses and Associates (ANOTE-ANIGEA) National Committee created a working group including 20 expert nurses and 2 physicians, divided into 4 working groups. Each group worked on one level of training: trainee endoscopy nurse, competent endoscopy nurse, advanced endoscopy nurse, and nontechnical skills. For the final declaration, the Delphi method was adopted. This position paper defined the role and training of endoscopy nurses, proposing three progressive levels of professional preparation. This is the first document aimed at defining the role and training of endoscopy nurses, suggesting progressive levels of professional preparation. It could be an appropriate tool for both nurses who are beginning to work in an endoscopic unit, as well as for all subjects involved in training nurses practicing in digestive endoscopy.

As globally recognized, the role of the endoscopy nurse encompasses all stages of endoscopic procedures, from before examination to discharge, taking care of patients by providing relational, technical, and educational assistance. Indeed, nurses help the patient to ensure privacy, respect for dignity, comfort, and safety by applying specific theoretical knowledge and practical skills (ESGENA, 2004, 2008; Verschuur et al., 2007). This complex task demands continuous professional development to provide holistic care for patients with a variety of gastrointestinal diseases (Tucker et al., 2017). However, different structured training pathways in this specific field are available in different countries (Tucker et al., 2017), and the minimal standards and definition of training levels for endoscopy nurses are not clearly defined worldwide.

## Background

The educational and training pathways for endoscopy nurses present significant disparities across diverse countries (Tucker et al., 2017). For instance, in the UK (Drew et al., 2013; Sprout, 2000), due to the lack of physician reimbursement for these procedures, endoscopy nurses have shouldered wider responsibilities, prompting the development of a comprehensive educational model tailored for these expanded roles. The measurable skills of endoscopy nurses include assessing learning needs, providing detailed responses to disease-related inquiries, and delivering health education. This emphasizes the importance of cognitive abilities such as understanding patient risk and recommending appropriate procedures and treatments. In addition, eight core competencies for endoscopy nurses are considered, encompassing emergency care, patient monitoring, and infection control. These competencies suggest that quality care provision by endoscopy nurses requires a broad range of procedural, cognitive, and interpersonal skills.

In some Asian countries, such as China and Hong Kong, highly structured training programs for endoscopy nurses are established (Ren et al., 2019). The Society for Gastroenterology in Hong Kong has developed an evidence-based six-month training program incorporating both theoretical and practical teachings (Ren et al., 2019). In the United States, the Society of Gastroenterology Nurses and Associates (SGNA) (Calderwood et al., 2018; Wright, 2000) provides guidance for a thorough preparation of endoscopy nurses, facilitating high standards of patient care. Indeed, the SGNA document underscores stringent cleaning and sterilization procedures for equipment, safe medication administration practices, adherence to infection control guidelines, optimal staffing levels, patient monitoring, proper storage, and medication procedures as integral components of patient safety.

Based on these considerations, a document appropriately defining the minimum requirements, standards, and advanced levels of competence and role for the digestive endoscopy nurse was recommended (Duncan et al., 2017; Duffield et al., 2017; Mahawongkajit et al., 2022). In Italy, the mission of ANOTE-ANIGEA (National Association of Operators of Endoscopic Techniques – National Association of Gastroenterology Nurses and Associates) is to promote training, professional update, and scientific research for endoscopy nurses and technicians. Therefore, an official document from ANOTE-ANIGEA defining the skills and roles of endoscopy nurses was worthwhile. Following a literature review, a worldwide universal endoscopy nurse training program could not be found. Some countries had better documented training programs than others. They looked at theoretical knowledge as well as practical skills in all phases that a nurse works during a patient’s endoscopy visit. A consensus process was used by ANOTE-ANIGEA to identify domains for skills definitions. As a result, three levels of endoscopy nurse training were proposed (Trainee Endoscopy Nurse, TEN; Competent Endoscopy Nurse, CEN; Advanced Endoscopy Nurse, AEN) to outline the skills and roles of each level.

## Methods

### Consensus Process

The ANOTE-ANIGEA National Committee designed a working group that included associated nurses with appropriate knowledge and skills. The consensus process was formulated between July 2021 and December 2021, according to the following steps: (a) identification of the key issues to be developed; (b) selection of experts in endoscopy; and (c) formation of different working groups (WG). Each component of the WGs was required to prepare different statements and achieve consensus using the Delphi method (Hsu and Sandford, 2007).

A final face-to-face meeting was then held to share the statements of the WGs and outline the first draft of the manuscript. The final experts included 22 members (20 nurses and 2 physicians), divided into three WGs to define the roles and skills of endoscopy nurses, identifying the following three levels of training: (1) trainee endoscopy nurse (first level); (2) competent endoscopy nurse (second level); and (3) advanced endoscopy nurse (third level). Definitions of non-technical skills were also provided. In January 2022, the elaborated statements were uploaded to an online voting system (http://scott.armstrong.delphi.stlouisintegration.com/delphi2/) and distributed to each WG. For each statement, the participants were asked to rate their level of agreement: (1) strongly agree, (2) agree, (3) equivocal, (4) disagree, and (5) strongly disagree.

Data were collected, addressed, and shared with all experts after each review round. Consensus was achieved when at least 80% of the respondents expressed “strong agreement” or “agreement” with each statement. Statements that did not reach this threshold were revised, modified, and rated again in subsequent rounds of voting until a final agreement was reached. Panel experts gathered in Rome on June 25, 2022 for refinement and final approval of the overall statement.

### Domains for Skills Definition

The panel proposed to evaluate the training level grading the subsequent seven domains using the following scores: (0) insufficient; (1) poor; (2) sufficient; (3) adequate; (4) good; (5) very good; and (6) excellent.

#### 1. Specialized knowledge and technical skills

The endoscopy nurse should appropriately know nursing theory, pathophysiology of the digestive system,foundational clinical practice, and endoscopic devices and techniques, including complex procedures (Riegert et al., 2020).

#### 2. Nursing assessment and intervention skills

The endoscopy nurse should be able to plan nursing care with other members of the healthcare team.The nurse identifies care goals, formulates the patient care plan, and evaluates results. The nurse should be able to recognize emergency/urgent situations and implement actions aimed at directing patient assistance. The endoscopy nurse ensures continuity of patient care during the pre-, intra-, and post-procedure phases, including early recognition of complications (Dokoutsidou et al., 2020).

#### 3. Health, safety, public hygiene, and cancer screening

The endoscopy nurse must implement measures to promote health and care for patients. Nurses set up the endoscopic room before each procedure, checking the presence and correct functioning of medical devices and electromedical equipment. The nurse should be able to perform the pre-procedure assessment (collection of the relevant medical history and nursing records), ensuring that the path of the patient is safe and respects privacy until discharge (Bisschops et al., 2021). She/he should be aware of colorectal cancer prevention programs and able to inform and educate patient on the appropriate procedure for cancer detection and follow-up. The nurse should know radiological risk and primary measures for radioprotection (Gartlehner et al., 2023).

#### 4. Control of infection in the endoscopic environment

The endoscopy nurse actively participates in the prevention of healthcare-related infections. The nurse assures compliance with hand washing and with specific indications on the use of personal protective equipment (Calderwood et al., 2018).

#### 5. Knowledge and care of endoscopic equipment

The endoscopy nurse should know the endoscopic instruments and devices, and verify their functioning and setting for specific programmes of electromedical and electrosurgical equipment. The nurse should be able to handle and use endoscopic instruments correctly, perform the pre-and post-procedure leak test, and promptly identify any malfunctions and/or damage. Nurses should know the correct functioning of the scope washing machine, washing pump, and storage cabinet, and ensure the traceability of the various steps (Beilenhoff et al., 2018).

#### 6. Professional and ethical practice

The endoscopy nurse is responsible for training and should comply with the official policies, guidelines, and procedures in the service area.The nurse guarantees the rights and duties of the patient. The nurse maintains patient confidentiality by recognizing and upholding patient rights and privileges and ensuring patient safety (Ladas et al., 2007).

#### 7. Commitment to the development of professional practice

The endoscopy nurse constantly maintains and updates knowledge to provide quality care and refers to studies based on scientific evidence (evidence-based nursing). The nurse should share innovations with the team, participate in scientific society initiatives, and promote nursing research (Manno et al., 2021).

## Statement

***Statement 1*:** The TEN (first level) is a professional in the endoscopy unit, who requires specific support and tutorial from an expert endoscopy nurse.

The trainee is either a newly graduated nurse or with experience in other nursing specialities. At the end of the training period, he should acquire skills for collaboration in low-complexity diagnostic and therapeutic procedures and be autonomous in dealing with instruments and device reprocessing. The nurse can assist patients in various phases of sedation-analgesia. The trainee nurse correctly manages the histological samples, from collection in suitable containers and safe storage, until transport to the pathology laboratory. The trainee nurse can participate in microbiological surveillance by performing endoscope sampling after specific preparation.

Endoscopy nurses should be aware of the prevention and screening programmes for colorectal cancer and be able to give summary indications in this field. However, the TEN still requires appropriate support and adequate training to be involved in more complex endoscopic procedures, and achieve more knowledge on the most frequent gastrointestinal diseases and the main endoscopic procedures.

**FIGURE 1.**
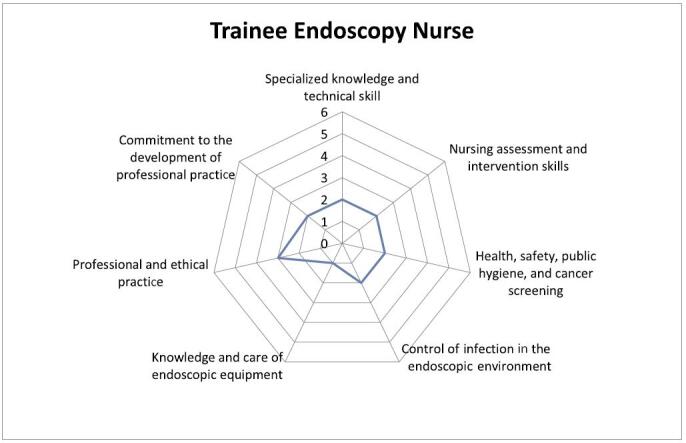
Trainee endoscopy nurse.

The minimum scores to be achieved by a trainee endoscopy nurse are as follows (Figure 1):

- Specialized knowledge and technical skills: 2
- Nursing assessment and intervention skills: 2
- Health, safety, public hygiene, and cancer screening: 2
- Control of endoscopic environment infection: 2
- Knowledge and care of endoscopic equipment: 1
- Professional and ethical practice: 3
- Commitment to the development of professional practice: 2

***Statement 2*:** The competent endoscopy nurse (second level) is adequately trained and has acquired the knowledge and skills necessary to assist the patient and endoscopist during complex endoscopic procedures.

The CEN should know the primary diseases of patients referred for digestive endoscopy, the more complex endoscopic procedures, and how to prevent and manage complications by preparing the patient throughout the periprocedural process. The nurse autonomously performs her care activities and collaborates with the working team. This nurse should know nursing procedures in digestive endoscopic emergencies, including those related to hemostasis of gastrointestinal bleeding and endoscopic dilation. Following appropriate training, the nurse can care for patients with artificial nutrition and nutritional support. The CEN is available to tutor the trainee nurse.

**FIGURE 2.**
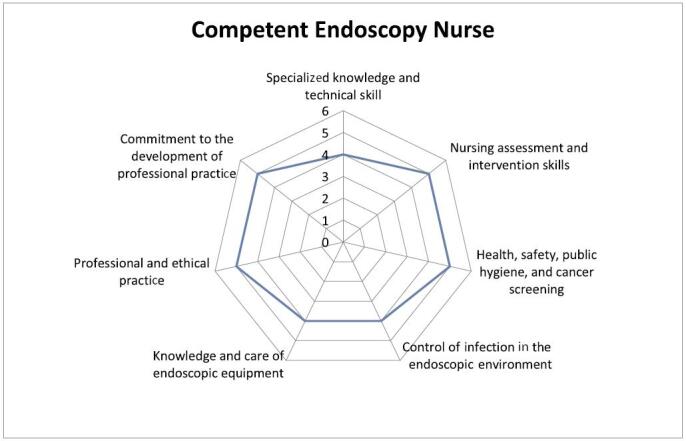
Competent endoscopy nurse.

The minimum scores to be achieved by a competent endoscopy nurse are as follows (Figure 2):

- Specialized knowledge and technical skills: 4
- Nursing assessment and intervention skills: 5
- Health, safety, public hygiene, and cancer screening: 5
- Control of infection of the endoscopic environment: 4
- Knowledge and care of endoscopic equipment: 4
- Professional and ethical practice: 5
- Commitment to the development of professional practice: 5

***Statement 2a*:** The CEN knows the procedural sedation-analgesia administered to facilitate the execution of diagnostic/therapeutic endoscopic procedures without discomfort for the patient.

Procedural sedation-analgesia consists of the administration of a hypnotic and/or analgesic to facilitate performance of the diagnostic or therapeutic endoscopic procedure without (or with minimal) discomfort to the patient. Procedural sedation-analgesia is included among the quality indicators for endoscopic procedures. Sedation implies a condition of reduced level of consciousness, which may be assessed by the Richmond Agitation Sedation Scale. Sedation can progress or regress from one level to another, resulting in an inevitable variation in risk that deserves constant evaluation to ensure patient safety. Therefore, procedural sedation-analgesia requires careful monitoring of vital parameters to prevent potential adverse events and the nurse administering it should be specifically trained (Dossa et al., 2021).

***Statement 2b*:** The CEN collaborates with the physicians (endoscopist, anesthetist) during endoscopic emergency procedures in the upper and lower digestive tracts.

Endoscopic emergencies in the upper tract include mainly hemorrhages, impacted food in the esophagus, foreign body or caustic ingestion, and cholangitis (Chirica et al., 2019; Kate et al., 2022). In the lower tract, endoscopic urgency is essentially related to hemorrhage after endoscopic therapeutic procedures (polypectomies, mucosal resection, etc.), surgical interventions (anastomotic leak), or to resolve a colon volvulus (Hollenbach et al., 2021). The nurse collects clinical information, with particular attention to diseases and ongoing antithrombotic therapy (anticoagulant and antiaggregant).The nurse evaluates the state of consciousness and ventilation, which are essential for preventing the risk of pneumonia aspiration and guarantees the comfort of patients.

The nurse should know the procedures and devices used by the anesthetist. The CEN also knows emergency endoscopic procedures and prepares the appropriate devices based on the potential needs. The nurse collaborates with the endoscopist during the procedure in the use of devices or drugs and provide adequate assistance in the post-procedure phase to promptly disclose any potential complications.The nurse care for the patient (when required), assesses the state of consciousness, ventilates the patient (when required), and collaborates with the anesthetist during tracheal intubation, knowing the devices and drugs used.

***Statement 2c*:** The CEN supports the endoscopist in performing all procedures for endoscopic hemostasis.

The CEN should know the most common causes of bleeding in both the upper gastrointestinal tract (ulcers, erosions, esophago-gastric varices, Mallory-Weiss or Dieulafoy lesions, and neoplasms), and the lower tract (angiodysplasias, diverticula, colitis, neoplasms, radiation proctitis and sites of recent polypectomy).The nurse is aware of the procedures and devices for injection, mechanical, thermal, and topical techniques used for endoscopic hemostasis. The nurse collaborates with the endoscopist to prepare solutions (adrenaline, cyanoacrylate, polidocanol, hemostatic powders), position endoscopic clips (Endoclip, over-the-scope clip) or band ligation system, and set up an argon-plasma coagulation device (Gralnek et al., 2022). The competent endoscopy nurse provides adequate health education to the patient and caregivers about how to recognize the signs of potential complications after the procedure.

***Statement 2d*:** The CEN collaborates with the endoscopist to perform percutaneous endoscopic gastrostomy (PEG).

Percutaneous endoscopic gastrostomy (PEG) represents a valid therapeutic approach in patients with a benign prognosis in which there is a mechanical or functional impediment to the correct passage of the food bolus from the mouth to the stomach. PEG can also be useful when gastrointestinal decompression, rehydration, or administration of some drugs is necessary. The positioning of the nutritional probe is generally performed in the “pull” mode, with endoscopic introduction from the mouth to the stomach, where it is captured through a small incision in the abdominal wall (Choi and Cho, 2022). Nurses collaborate with the endoscopist during the procedure and with the multidisciplinary team to educate the patient and caregivers about device use, supporting other nursing staff for any complications.

***Statement 3*:** The advanced endoscopy nurse (third level) is adequately trained to work in endoscopic centers where complex diagnostic and therapeutic procedures are performed.This nurse is active in the training of other nurses and nursing research.

The AEN can support the endoscopist in performing endoscopic retrograde cholangiopancreatography (ERCP) and ultrasound endoscopy (EUS). She is trained in different operative endoscopic techniques (ablative, resective, dilation, stenting, bariatric endoscopy, etc.) or specific diagnostic (enteroscopy, videocapsule endoscopy, digestive pathophysiology, etc.) procedures. When specifically trained, she can act as an inflammatory bowel disease (IBD) nurse. The nurse is aware of the risks associated with radiological exposure and radioprotection.The nurse is able to train and tutor less expert nurses and is involved in nursing research. The nurse provides adequate health education to the patient or any caregiver about nutritional behavior, screening programs, and follow-up. Since not all procedures are performed in each endoscopy unit, the nurse can develop varied advanced skills.

**FIGURE 3.**
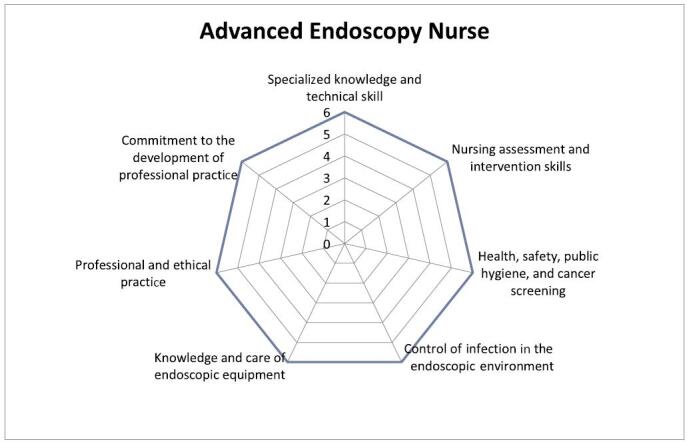
Advanced endoscopy nurse.

The scores to be achieved by the advanced endoscopy nurse are as follows (Figure 3):

- Specialized knowledge and technical skills: 6
- Nursing assessment and intervention skills: 6
- Health, safety, public hygiene, and cancer screening: 6
- Control of infection in the endoscopic environment: 6
- Knowledge and care of endoscopic equipment: 6
- Professional and ethical practice: 6
- Commitment to the development of professional practice: 6

***Statement 3a*:** The AEN can support the endoscopist in performing endoscopic retrograde cholangiopancreatography (ERCP).

ERCP is a procedure used for the diagnosis and treatment of some benign or neoplastic lesions of the bile ducts, gallbladder, and pancreas. ERCP consists of cannulation of the bile ducts using the wire-guided technique with a sphincterotome, and injection of contrast medium to visualize the bile ducts. Different therapeutic procedures (sphincterotomy, gallstone removal, dilation, stent positioning) can then be performed. The AEN knows all devices (guide-wire, stones extraction basket, balloons, stents, etc.), and is able to collaborate with the endoscopist in all procedures (Johnson et al., 2020).The nurse cares for the patient verifying that no complications occur in the post-procedure phase.

***Statement 3b*:** The AEN collaborates with the ERCP.

The AEN is able in performing all coordinated and integrated maneuvers with those of the endoscopist, has technical ability and experience in the use of different devices, and speed of action in changing accessories so that this nurse may act as the first assistant of the operator, when specifically credentialed by the hospital, during ERCP procedures, particularly when the second operator is not present (Johnson et al., 2020). The nurse is prepared in monitoring patients to detect early complications in the post-procedure phase until the patient is transferred to the department of origin.

***Statement 3c:*** The AEN collaborates with the endoscopist to perform ultrasound endoscopy (EUS).

EUS combines endoscopy and ultrasound. EUS is performed to visualize and sample lesions in the pancreas, gastrointestinal mucosa, mediastinum, and retroperitoneum. Sampling procedures are performed by fine needle aspiration or fine needle biopsy, to perform a cytological or histological examination, respectively. EUS also allows therapeutic procedures, including the drainage of pancreatic or gallbladder collections, or abscesses, as well as injection of substances for the treatment of pancreatic masses, liver lesions, and celiac plexus neurolysis (Yousaf et al., 2020). The AEN knows how to prepare the endoscopy room, echoendoscope, and ultrasound device for EUS. The nurse prepares the most suitable accessories for the procedure, allowing the synergy of two different diagnostic techniques (endoscopic and ultrasound). In particular, the nurse knows how to handle the different biopsy needles based on diagnostic and therapeutic examinations and correctly collects and stores biological samples taken during the procedure.

***Statement 3d*:** The AEN is an expert at supporting the endoscopist in different ablative and resective procedures.

The currently used ablation technique is radiofrequency, primarily to treat Barrett’s esophagus, the precursor of esophageal adenocarcinoma. This consists of the use of a specific device through the endoscope placed in contact with the metaplastic dysplastic epithelium in the esophagus and causes cauterization. The treated pathological epithelium is then replaced by a normal esophageal epithelium during the regenerative phase. Different resection techniques are available, including endoscopic mucosal resection (EMR), endoscopic submucosal dissection (ESD) and full-thickness resection (FTR), as well as Zenker diverticulectomy (Moreira et al., 2023). The nurse knows how to handle these different devices and collaborates with the endoscopist in these procedures. The nurse can be prepared to assist the endoscopist during peroral esophageal endoscopic myotomy (POEM) to treat achalasia and other motility disorders.

***Statement 3e*:** The AEN can help the endoscopist in the treatment of benign or malignant stenosis of the intestinal and biliary tracts.

A stricture is a narrowing of the intestinal or biliary tract of a benign or malignant nature. Benign strictures develop after repeated or chronic inflammatory processes that cause thickening of the intestinal wall. Stricture can also occur after intestinal surgery in the area of the anastomosis or after resection or ablation endoscopic treatments (e.g., Barrett’s thermoablation, ESD in the esophagus) due to scar tissue formation.

Endoscopic dilation may be performed with mechanical or pneumatic techniques. Mechanical dilation is performed by endoscopically introducing coaxial dilators that slide on a the guidewire through the stenosis. Pneumatic dilation uses predetermined diameter dilator balloons which are introduced endoscopically over a guidewire and inflated through the stenosis under manometric control. Both dilation procedures may require radiological support. Dilation can also be achieved by positioning specially designed stents, particularly when the stenosis is neoplastic, in both the intestinal and biliopancreatic tracts (Nakai et al., 2020). The nurse knows all devices and is trained to collaborate with the endoscopist in all procedures. The nurse cares for the patient verifying that no complications occur in the post-procedure phase.

***Statement 3f*:** The AEN can assist the endoscopist during both diagnostic and therapeutic enteroscopy.

Enteroscopy is a procedure to study the small intestine. It is a lengthy (may take a few hours) and complex endoscopic examination, performed under deep sedation, using an enteroscope, a special instrument that is 200 cm long. The double balloon technique is the most widespread and the examination can be performed orally and rectally, thus allowing the entire small intestine to be visualized in most cases. However, due to its complexity, invasiveness, and the possibility of complications (perforation, pancreatitis), enteroscopy is performed mainly for diagnostic definition (biopsies) or treatment (hemostasis, polypectomy, dilation, etc.) of lesions highlighted by previous radiological investigations or small-bowel capsule endoscopy (Rondonotti et al., 2018; Tringali et al., 2021). The nurse knows the techniques for advanced enteroscopy and instruments. She also knows all devices and is prepared to collaborate with the endoscopist in this procedure.

***Statement 3g*:** The AEN is involved in the study of the small intestine with small bowel capsule endoscopy (SBCE).

SBCE is a disposable device that allows for study of the small intestine. The capsule is swallowed by the patient and sends the images in real time to a recorder through detector electrodes or a band applied to the abdomen. The capsule progresses via intestinal peristalsis and is generally expelled in 24–48 hours. The nurse provides counseling before the SBCE examination, giving explanations to the patient about how the investigation is performed and the related preparation of the bowel. The nurse can verify capsule arrival in the stomach by viewing it on the recorder. Upon return of the recorder, the nurse can prepare the video and verify that the SBCE has passed through the cecum to confirm that the study was completed (Ionescu et al., 2022). The appropriately trained nurse can perform a pre-reading of the records, highlighting suspicious images that are subsequently more easily evaluated by the physician, who is responsible for the final diagnosis.

***Statement 3h*:** The AEN collaborates with the clinician during manometry procedures and high-resolution impedance pH monitoring.

High-resolution esophageal manometry is a test that measures peristalsis and pressure of the lower esophageal sphincter using a catheter inserted into the esophagus through the nostril. It allows the diagnosis of the main disorders of intestinal motility such as achalasia, diffuse esophageal spasm, and Jackhammer esophagus. Anorectal manometry is a test that measures the pressure of the anal sphincter, sensitivity of the rectum, and coordination between the rectal and anal muscles. A small catheter with a balloon at the end is inserted into the rectum and inflated to establish pressures. The procedure is performed to treat disorders related to evacuation, such as pelvic floor syndrome (obstructed defecation syndrome, puborectal sling syndrome, etc.). For these procedures, the nurse is able to inform and instruct the patient on the examination, collaborate in positioning and maintaining the correct positioning of the probes and medical equipment during the investigation, and ensure patient comfort and privacy during the examination.

24-hour pH-impedance monitoring allows detection and recording of the pH values of liquid or gaseous reflux in the esophagus by using a long electrode introduced through the nostril and connected to a portable recorder (Savarino et al., 2019).The advanced endoscopy nurse collaborate with the physician to insert the device and inform the patient of the subsequent 24-hour behavior.

***Statement 3i*:** The AEN is an expert in nursing assistance for patients with IBD.

IBD include ulcerative colitis and Crohn’s disease. The AEN knows the main diagnostic tests and therapies and is familiar with the endoscopic classification. Patients with IBD undergo colonoscopy several times, so it is of fundamental importance to create dedicated endoscopic care pathways in collaboration with IBD Units (Lamb et al., 2019). The nurse should know the most frequent complications of disease, such as perianal fistulas and abscesses, as well as the effects of surgical therapy and the surgical implications of the postoperative endoscopic approach. Nurse is prepared to work with the endoscopist in all procedures and knows all devices and procedures.

***Statement 3l*:** The AEN assists the endoscopist in the drainage of pancreatic and gallbladder collections.

The drainage of pancreatic or gallbladder collections is performed by ERCP or ultrasound endoscopy by placing plastic or metal stents. Pancreatic collection generally occurs after pancreatitis, while gallbladder drainage is required for acute cholecystitis resistant to medical therapy in patients at high surgical risk or inoperable (DeSimone et al., 2017). In the latter case, using a metallic stent, it is possible to create a passage between the wall of the collection and the intestinal lumen (duodenum or stomach), avoiding surgery.

***Statement 3m*:** The AEN collaborates with the endoscopist during various procedures of bariatric endoscopy.

The prevalence of obesity is increasing around the world. Bariatric endoscopy consisted first of insertion of intragastric balloons to reduce appetite. During the past decade, numerous techniques have been introduced, including the trans-pyloric shuttle, endoscopic sleeve gastroplasty, primary obesity, endoluminal surgery, and aspiration therapy (Dang et al., 2023). During these minimally invasive therapeutic approaches, various endoscopic accessories are used that the advanced endoscopy nurse should be able to handle.

***Statement 3n*:** The AEN collaborates with the clinician for fecal microbiota transplantation (FMT).

FMT is a procedure consisting of inoculation of a stool supernatant from a healthy donor to a recipient patient. This therapeutic approach was introduced mainly for the treatment of antibiotic-resistant or recurrent *Clostridium difficile* infections, with extraordinary results. The product used is properly prepared in bags and stored in biobanks. The bags are thawed and administered to the recipient by colonoscopy, gastroduodenoscopy, or via a naso-jejunal tube (Lopetuso et al., 2023). The AEN knows the screening tests necessary for both the patient and the donor to perform FMT.The nurse helps the patient identify the potential donor, usually among family members or friends, and the endoscopist during inoculation.

***Statement 3o*:** The AEN is involved in nursing and medical research.

Nurses with specific skills can participate in experimental trials of both medical devices and drugs. Nurses can select and coordinate suitable patients for the study and manage their follow-up, ensuring accurate data collection. The nurse can plan, participate, and conduct clinical or experimental studies on nursing topics in the endoscopic and gastroenterological field. Nursing research is fundamental to increase scientific evidence on different topics of work and to maintain professional skills (Eckardt et al., 2017; Ryder and Jacob, 2022).

***Statement 4:*** The endoscopy nurse must acquire non-technical skills (NTS).

NTS are necessary to obtain effective results when interacting with team members, patients, and families (Hitchins et al., 2018). They represent the ability to approach healthcare settings and organizational dynamics that can evolve rapidly. The acquisition of these skills, which requires time and experience in the sector, is a great support for specific technical skills. NTSs are often overlooked or poorly implemented during studies and professional training because they are not immediately perceived, considered “innate,” and are passed down informally. However, the development of NTS is essential to minimize errors, avoid adverse events, and ensure an efficient and safe working environment, providing the best quality of care. The three domains in which NTSs are identified are (a) communication, (b) teamwork and leadership, and (c) awareness of the work environment.

***Statement 4a:*** Communication.

Knowledge is the result of the transmission of information, thoughts, emotions, and skills that every nurse should develop during the profession by working in a team, and should be able to provide the most accurate information to the patient, guaranteeing safety and comfort, and providing clear and comprehensible information. Communication must be clear, accurate, complete, and timely to be effective and must overcome difficulties resulting from lack of time, linguistic problems, or cultural and social barriers (Newell & Jordan, 2015).

***Statement 4b:*** Teamwork and leadership.

Working in a digestive endoscopy service can be complex and tiring. Therefore, the presence of a guide figure is essential to achieve an effective and efficient working context. Optimal leadership motivates staff to collaborate, stimulates the deepening of their knowledge, and promotes conditions for a more serene working environment, which brings greater satisfaction perceived by patients (Major, 2019; Pettit & Duffy, 2015).

***Statement 4c:*** Awareness of the working environment.

Interventions designed to improve teamwork and communication can have beneficial effects on technical performance and patient outcomes, including reduction of errors. Specifically, the endoscopy room is a complex place with many potential factors that can interfere with endoscopic procedures and predispose to negative events. Optimizing the operating environment can have an impact on overall endoscopic results, improving individual endoscopic skills. Therefore, all team members should recognize the importance of adequate procedure planning and contribute effectively to strategies to optimize endoscopic activity (Bayramzadeh et al., 2021).

## Conclusions

Undeniably, the roles of a nurse working in the endoscopy unit have become increasingly complex, both for professional skill and legal implications. Several endoscopic procedures, techniques, and devices are relentlessly introduced in clinical practice, so that continuous theoretical and practical training are needed. This is the first document aimed at defining the role and training of endoscopy nurses proposing three progressive levels of professional preparation (see Supplemental Digital Content Table 1, available at: http://links.lww.com/GNJ/A126). It could be an appropriate tool for both nurses who are beginning to work in an endoscopic unit, as well as for all operators involved in training nurses in the digestive endoscopy specialty. Finally, the implementation of this document could be particularly useful in those countries where the “endoscopy nurse” is still not appropriately recognized by institutions as a “specialist nurse.”
